# Genetic, clinical, and biochemical profiling of Gilbert syndrome in a Nepali cohort: High prevalence of the UGT1A1 c.-3279T>G polymorphism and correlation with hematological parameters

**DOI:** 10.1371/journal.pone.0347128

**Published:** 2026-04-13

**Authors:** Pragya Gautam Ghimire, Prasanna Ghimire, Rajan Pande

**Affiliations:** 1 Department of Pathology, Nepalgunj Medical College and Teaching Hospital, Banke, Nepal; 2 Department of Radiology, Nepalgunj Medical College and Teaching Hospital, Banke, Nepal; 3 Department of Internal Medicine, Bheri Hospital, Banke, Nepal; Fudan University, CHINA

## Abstract

**Introduction:**

Gilbert Syndrome (GS) is a common hereditary disorder characterized by intermittent jaundice. The pathogenesis is unconjugated hyperbilirubinemia due to reduced hepatic UDP-glucuronosyltransferase 1A1 (UGT1A1) activity. Its genetic basis relies on c.-3279T > G polymorphism (UGT1A1*60), which reduces gene transcription by approximately 40%, and is highly prevalent in Asian populations.

**Aims:**

This study aimed to profile the genetic, biochemical, and clinical characteristics of individuals with clinical features of GS in Nepal and examine correlations between UGT1A1 genotypes and hematological parameters.

**Methods:**

This study utilized a prospective descriptive design supplemented by a retrospective review of medical records, including 75 patients with isolated unconjugated hyperbilirubinemia. Prospective recruitment and data collection were conducted from July 18, 2025, to November 30, 2025. Medical records from outside facilities were accessed for research purposes from July 18, 2025, to November 30, 2025, covering records dating back to January 1, 2021. Patients underwent ARMS-PCR genetic testing for the UGT1A1 c.-3279T > G variant.Patients with hemolysis or hepatobiliary disease were excluded. Genetic confirmation of GS was based on the presence of the G allele.

**Results:**

Mean age of cohort was 28.9 ± 10.4 years (range: 14–55), with a male predominance (69.3%). Genotype distribution revealed 66.7% homozygous mutant (G/G), 29.3% heterozygous (G/T), and 4% wild-type (T/T), yielding a G allele frequency of 81.3%. Mean bilirubin levels showed a genotype-phenotype correlation: G/G (4.3 ± 1.1 mg/dL), G/T (3.2 ± 0.9 mg/dL), and T/T (2.4 ± 0.3 mg/dL). Hematological parameters were within normal reference ranges across all genotypes, confirming the non-hemolytic nature of the condition. No hepatosplenomegaly was detected on ultrasonography.

**Conclusion:**

This study demonstrates an exceptionally high prevalence of the UGT1A1*60 G allele among individuals with clinical features of GS in Nepal. These findings reaffirm the benign, non-hemolytic character of GS and underscore the diagnostic and pharmacogenetic utility of UGT1A1 genotyping in the Nepali population.

## Introduction

Gilbert Syndrome (GS), affects 3–10% population globally and is the most common hereditary disorder of bilirubin metabolism [1]. It presents with intermittent jaundice which shows mild, unconjugated hyperbilirubinemia (typically <6 mg/dL), arising from reduced UGT1A1 enzyme activity essential for bilirubin conjugation during lab investigation [2, 3]. However, abnormal liver function and hemolysis are all absent. UGT1A1 deficiency disorders represent a phenotyping continuum ranging from the benign hyperbilirubinemia of Gilbert syndrome to the severe neonatal jaundice of Crigler – Najjar syndrome Types I and II which highlights importance of accurate genetic characterization across this spectrum. Studies on genetic variants show population specificity: Western Caucasians predominantly carrying UGT1A128 (extra TA repeat reducing transcription), while Asians harboring UGT1A160 (c.-3279T > G), impairing transcription by ~40% [4–6]. This knowledge gap impacts clinical care as accurate genetic profiling prevents unnecessary testing and identifies patients at risk for drug toxicity from irinotecan and atazanavir, which require dose adjustment in UGT1A1 deficiency [7,8]. Despite presumed 3–20% South Asian prevalence, the Nepalese data remain sparse.7 This study provides the first comprehensive genetic and biochemical characterization of Nepali GS patients, genetically examining UGT1A1 c.-3279T > G prevalence, genotype-phenotype correlations, and hematological associations confirming non-hemolytic hyperbilirubinemia.

## Methods

### Study design and participants

This study utilized a prospective descriptive design, supplemented by a retrospective review of medical records for patients who provided prior laboratory reports and reports from outside facilities. Prospective recruitment and data collection were conducted from July 18, 2025, to November 30, 2025. Medical records and laboratory data from outside facilities were accessed and reviewed for research purposes from July 18, 2025, to November 30, 2025, covering patient records dating back to January 1, 2021.

### Inclusion criteria

Patients presenting with isolated unconjugated hyperbilirubinemia. Definitive inclusion required:

- Total serum bilirubin >2.0 mg/dL
- Direct (conjugated) bilirubin <0.5 mg/dL (or <20% of total bilirubin)
- Absence of overt clinical signs of hemolysis
- No laboratory signs of hemolysis, including peripheral smear examination and reticulocyte counts.
- Genetic confirmation of the UGT1A1 c.-3279T > G polymorphism by ARMS-PCR

### Exclusion Criteria

Patients were excluded if they had:

- Laboratory evidence of hemolytic anemia (abnormal peripheral smear, reticulocyte count, and elevated LDH)
- Abnormal liver function tests suggesting hepatocellular disease
- Hepatosplenomegaly on ultrasonography
- History of chronic liver disease or biliary obstruction
- Recent history of drug intake that could cause hyperbilirubinemia
- Incomplete genetic or biochemical data

Although the primary inclusion criterion was the presence of the c.-3279T > G polymorphism, three prospectively enrolled patients with the wild-type T/T genotype were retained in the analysis for comparative purposes, as they met all clinical and biochemical criteria for GS at presentation. Their inclusion enabled genotype-phenotype correlation and served as a reference group for evaluating the gene-dosage effect. The biochemical- genetic discordance in these cases likely reflects rarer UGT1A1 coding or regulatory variants not captured by the two-variant genotyping pane, uncommon promoter alleles such as UGT1A1*36 or non- UGT1A1- mediated mechanisms which is a limitation inherent to targeted genotyping panels in resource-limited setting where comprehensive next-generation sequencing is not yet routinely available.

### Laboratory and genetic testing

Blood samples were collected in different serum and EDTA tubes from all the patients. Comprehensive biochemical and hematological investigations were performed including:

- Total and direct serum bilirubin measured using the diazo method
- Liver function tests: ALT, AST, and ALP measured by standard enzymatic assays
- Complete blood count (CBC), including hemoglobin, total and differential white blood cell count, and platelet count
- Peripheral blood examination and Reticulocyte count.

All biochemical analyses were performed using automated analyzers with appropriate quality control measures. Normal reference ranges were established based on the laboratory’s internal validation studies and were consistent with international standards. Biochemical profiles were established through current laboratory analysis or verified via retrospective review of authenticated past medical reports brought by the participants to confirm a history of isolated unconjugated hyperbilirubinemia.

Samples on EDTA tube were also subjected to genetic testing where Genomic DNA was extracted using standard phenol-chloroform extraction methods. Genomic DNA extraction and ARMS-PCR [2] analysis were performed at Decode Genomic and Research Center, an accredited external laboratory, as in-house molecular genotyping facilities are not available at our primary center. The genotyping panel included two established UGT1A1 polymorphisms: the c.-3279T > G promoter variant (UGT1A1*60) and the UGT1A1*28 TA-repeat (A(TA)7TAA) polymorphism.

### Radiological investigation

Abdominal ultrasonography was performed on all patients using a Logiq P6 ultrasound machine with a 3.5–5 MHz convex probe to systematically exclude hepatosplenomegaly, gallstones, biliary tract abnormalities, or other structural liver pathology that might contribute to hyperbilirubinemia.

### Ethical considerations

The study protocol was reviewed and approved by the Institutional Review Committee (IRC) of the Nepalgunj Medical College and Teaching Hospital (**Ref. No: 02/082–083**) on **July 18, 2025**. Written informed consent was obtained from all participants, or from parents/guardians in the case of minors. While the authors had access to identifying information during the collection of prospective samples and the review of past medical reports to ensure data integrity, all data were fully anonymized during the analysis phase to maintain participant confidentiality.

### Statistical analysis

Descriptive statistics were computed using Microsoft Excel 2016. Genotype and allele frequencies for the UGT1A1 c.-3279T > G polymorphism were determined by the direct count method. The Hardy-Weinberg equilibrium was assessed to ensure the genetic validity of the observed genotype distribution. Comparisons of mean bilirubin levels across different genotype groups were performed descriptively. Statistical significance was set at p < 0.05 for all analyses.

Bilirubin values are reported in mg/dL throughout. Where refrenced international studies reported values in μmol/L, conversion was applied using the standard factor of 1 mg/dL = 17.1 μmol/L.

## Results

### Demographic characteristics and clinical profile

The study population comprised 75 genetically confirmed Gilbert Syndrome patients with a mean age of 28.9 ± 10.4 years (range: 14–55 years). There was male predominance (n = 52, 69.3%) compared to females (n = 23, 30.7%), yielding a male-to-female ratio of 2.3:1. The age distribution analysis revealed that 65% of patients were within the 20–35-year age range consistent with the typical presentation pattern of GS during young adulthood. This syndrome in our study was also identified through incidental laboratory findings and during episodes of physiological stress.

### Molecular genetic analysis: genotype distribution and allele frequencies

Molecular analysis of the UGT1A1 c.-3279T > G polymorphism revealed a remarkably high prevalence of the mutant G allele within this Nepalese cohort. The genotypic distribution demonstrated marked deviation from patterns typically observed in Caucasian populations, with the following frequencies:

- Homozygous mutant genotype (G/G): 50 patients (66.7%)
- Heterozygous genotype (G/T): 22 patients (29.3%)
- Wild-type homozygous genotype (T/T): 3 patients (4.0%)

The demographic and genotypic characteristics of the study population are summarized in Table 1.

**Table 1:** Demographic and Genotypic Characteristics of Study Population (N = 75).

Parameter	Value
Age (years), mean ± SD	28.9 ± 10.4
Age range (years)	14-55
Male, n (%)	52 (69.3)
Female, n (%)	23 (30.7)
Male: Female ratio	2.3:1
**Genotype Distribution**	
G/G (Homozygous Mutant), n (%)	50 (66.7)
G/T (Heterozygous), n (%)	22 (29.3)
T/T (Wild-Type), n (%)	3 (4.0)
**Allele Frequency**	
G allele frequency	0.813 (81.3%)
T allele frequency	0.187 (18.7%)

**Fig 1:**
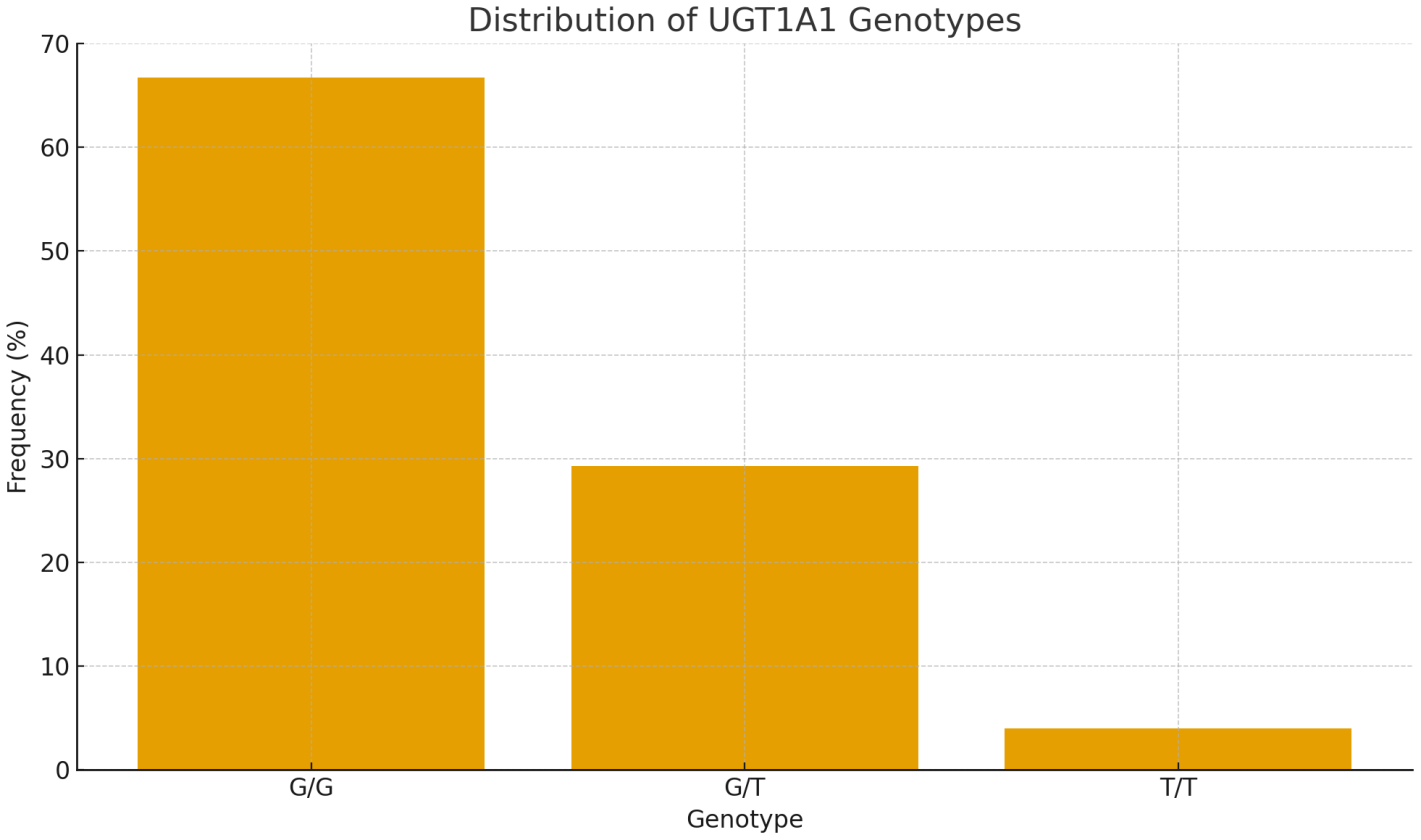
Distribution of UGT1A1 Genotypes. Bar chart showing the frequency of UGT1A1 genotypes (G/G, G/T, T/T) among 75 study participants.

The allele frequency calculation, performed using the Hardy-Weinberg equation, yielded a G (mutant) allele frequency of 0.813 (81.3%) and a T (wild-type) allele frequency of 0.187 (18.7%) (Fig 1). The observed genotype distribution was found to be in Hardy-Weinberg equilibrium (χ² = 0.23, p > 0.05), confirming that the study sample represents a population in genetic equilibrium for this locus and validating the reliability of our sampling methodology. This exceptionally high G allele frequency substantially exceeds that reported in most other Asian populations and is markedly higher than in Caucasian populations where the UGT1A1*28* variant predominates, thereby confirming that UGT1A160 represents the principal genetic determinant of Gilbert Syndrome in the Nepalese demography. Although the primary objective was to characterize genetically confirmed GS cases, three individuals with the wild-type T/T genotype were retained in the analysis. These participants met all clinical and biochemical criteria for GS at presentation but were subsequently found to lack the c.-3279T > G variant. Their inclusion enabled comparative analysis of genotype-phenotype relationships and served as a reference group to delineate the gene-dosage effect of the G allele.

### Laboratory analysis

All 75 patients exhibited the pathognomonic biochemical profile of Gilbert Syndrome, characterized by isolated unconjugated hyperbilirubinemia with preservation of hepatocellular functions.

### Biochemical assessment

*Bilirubin Profile:* The bilirubin profile analysis revealed:

- Total serum bilirubin: range 2.1–5.6 mg/dL (mean ± SD: 3.9 ± 1.2 mg/dL)
- Direct (conjugated) bilirubin: consistently <0.5 mg/dL in all subjects
- Indirect (unconjugated) bilirubin: > 80% of total bilirubin in all cases
- Direct bilirubin fraction: < 20% of total bilirubin

*Hepatic Function Markers:* Comprehensive hepatic function assessment demonstrated preserved hepatocellular integrity across the entire cohort:

- Alanine aminotransferase (ALT): 28.4 ± 8.2 U/L (reference range: 10–40 U/L)
- Aspartate aminotransferase (AST): 26.7 ± 7.5 U/L (reference range: 10–40 U/L)
- Alkaline phosphatase (ALP): 82.3 ± 18.6 U/L (reference range: 40–120 U/L)
- Serum albumin: within normal limits (35–50 g/L) in all subjects
- Prothrombin time: normal in all subjects

These findings conclusively excluded hepatocellular dysfunction, cholestasis, or any alternative hepatobiliary pathology, thereby satisfying the essential diagnostic criteria for Gilbert Syndrome.

### Hematological assessment

Hemolytic processes were not detected as to the observed hyperbilirubinemia. Thus, analysis of hematological indices and hemolysis markers revealed:

*Complete Blood Count and Peripheral smear Analysis:*

- Hemoglobin concentration: 14.1 ± 1.4 g/dL (males: 14.6 ± 1.2 g/dL; females: 13.2 ± 1.3 g/dL)
- Total leukocyte count: within normal reference range (4,000–11,000/μL)
- Platelet count: within normal reference range (150,000–400,000/μL)
- Erythrocyte morphology: normal on peripheral blood smear examination with no evidence of spherocytes, schistocytes, or other pathological red cell forms

*Hemolysis Exclusion Markers:*

- Reticulocyte count: 1.2 ± 0.3% (reference range: 0.5–2.0%)
- Corrected reticulocyte count: within normal limits

Normal serum LDH concentrations further provide definitive evidence excluding hemolysis as a contributor to the unconjugated hyperbilirubinemia. These findings are of paramount importance in resource-limited settings where hemolytic disorders due to hemoglobinopathies, infectious and autoimmune diseases causing hemolysis are the first differential diagnoses.

### Radiological assessment

Systematic ultrasonographic evaluation of the hepatobiliary system was performed in all 75 subjects. None of the patient demonstrated: hepatomegaly (liver span>normal for age and gender); splenomegaly (splenic length >12.5 cm), hepatic parenchymal lesions, cholelithiasis or biliary sludge, intra- or extra-hepatic biliary ductal dilatation, portal vein thrombosis or alternative vascular abnormalities

### Genotype-phenotype correlation analysis

A statistically significant genotype-phenotype correlation was established between the UGT1A1 c.-3279T > G genotype and serum total bilirubin concentration, demonstrating a clear gene-dosage effect (Table 2). The analysis revealed a progressive, stepwise relationship between the number of mutant G alleles and the magnitude of hyperbilirubinemia, consistent with the predicted effect of reduced UGT1A1 enzymatic activity on bilirubin conjugation capacity.

**Table 2:** Genotype-Phenotype Correlation Analysis.

Genotype	Number of Patients (%)	Mean Total Bilirubin (mg/dL) ± SD	Range (mg/dL)	p-value*
G/G (Homozygous Mutant)	50 (66.7%)	4.3 ± 1.1	2.8-5.6	Reference
G/T (Heterozygous)	22 (29.3%)	3.2 ± 0.9	2.2-4.8	<0.001
T/T (Wild-Type)	3 (4.0%)	2.4 ± 0.3	2.1-2.7	<0.001

*p-value calculated by comparison with G/G genotype group.

The observed pattern demonstrates that homozygosity for the mutant G allele (G/G genotype) is associated with the highest mean bilirubin levels (4.3 ± 1.1 mg/dL), heterozygosity (G/T genotype) with intermediate levels (3.2 ± 0.9 mg/dL), and wild-type homozygosity (T/T genotype) with the lowest levels (2.4 ± 0.3 mg/dL). This stepwise decrement in bilirubin concentration, correlating inversely with the number of functional T alleles, unequivocally demonstrates the gene-dosage effect of the c.-3279T > G polymorphism on UGT1A1 transcriptional activity and resultant bilirubin conjugation efficiency.

Importantly, stratified analysis of hematological parameters across the three genotypic groups revealed no statistically significant inter-group differences in hemoglobin concentration, LDH levels, or reticulocyte counts. This finding provides compelling evidence that the observed variation in bilirubin levels is attributable solely to genotype-dependent differences in UGT1A1 enzymatic activity rather than any concurrent hemolytic process, and further confirms that the degree of hyperbilirubinemia in GS is directly determined by the genetic burden of the c.-3279T > G mutation independent of erythrocyte destruction.

## Discussion

Our study represents the first comprehensive genetic, biochemical, and clinical diagnosis of Gilbert Syndrome in a Nepalese cohort.

The systematic exclusion of hemolysis by thorough hematological evaluation is salient for resource-limited settings where hemoglobinopathies (thalassemia, sickle cell disease), autoimmune disorders, enzymopathies (G6PD deficiency), and infectious causes of hemolysis (malaria) constitute important differential diagnoses. Normal serum LDH and reticulocyte indices in our groups establishes the non-hemolytic etiology and confirms that bilirubin elevation derives exclusively from impaired UGT1A1-mediated conjugation [9,10]. The hematological investigations provide a cost-effective diagnostic framework applicable in settings with limited access to advanced genetic testing [11].

This study exceptionally reveals a high prevalence of the UGT1A1*60 (c.-3279T > G)* polymorphism with a G allele frequency of 81.3% being the highest among globally reported cases for this variant. The homozygosity rate of 66.7% and the observed genotype distribution in Hardy-Weinberg equilibrium confirm that UGT1A160 represents the predominant genetic determinant of GS in this population, contrasting markedly with Caucasian populations where UGT1A1*28 predominates.

The observed G allele frequency (81.3%) substantially also exceeds previously reported frequencies from other Asian populations, including Chinese (40–70%) [12], Japanese (55–65%) [13], and Korean (50–60%) [14] cohorts. This striking difference may reflect population-specific evolutionary pressures, genetic drift, or founder effects unique to Himalayan populations. The ethnic-specific distribution of UGT1A1 variants has critical implications for diagnostic algorithms; in South Asian populations, screening strategies should prioritize c.-3279T > G detection over TA repeat analysis for optimal diagnostic yield [15].

The G/G homozygotes exhibiting bilirubin levels 79% higher than T/T homozygotes signify the functional significance of the c.-3279T > G variant with the number of copies of a gene directly influences the amount of gene product. This polymorphism, located within the phenobarbital-responsive enhancer module (PBREM) of the UGT1A1 promoter, disrupts binding sites for the constitutive androstane receptor (CAR) and pregnane X receptor (PXR), reducing transcriptional activation by approximately 40% [16,17]. The progressive reduction in bilirubin conjugation capacity across genotypes (G/G < G/T < T/T) provides in vivo validation of this transcriptional mechanism [18]. While our study focused on c.-3279T > G, future investigations should examine potential linkage disequilibrium with A(TA)7TAA and coding region variants to comprehensively elucidate the genetic architecture of GS in Nepal.

The high UGT1A1*60* allele frequency (81.3%) carries profound pharmacogenomic implications. Patients with reduced UGT1A1 activity demonstrate impaired glucuronidation of medications including irinotecan and atazanavir, predisposing to severe toxicity. FDA recommends dose reduction of irinotecan in UGT1A1*28 homozygotes; whether analogous recommendations should apply to UGT1A1*60 carriers requires validation through prospective pharmacogenetic studies [19]. Nevertheless, given that 66.7% of our cohort are G/G homozygotes and 96% carry at least one G allele, these findings suggest that preemptive UGT1A1 genotyping may have potential value in identifying patients at risk of impaired glucuronidation of irinotecan and atazanavir. Prospective studies directly assessing drug exposure and adverse outcomes in UGT1A1*60 carriers are warranted before definitive pharmacogenetic recommendations can be issued for this population [20].

It is important to contextualize Gilbert syndrome within the broader spectrum of UGT1A1 deficiency disorders. These conditions span a phenotyping continuum from the benign unconjugated hyperbilirubinemia of GS to the severe, life-threatening jaundice of Crigler-Najjar syndrome (CNS) Types I and II. Patients with CNS Type II may exhibit partial residual UGT1A1 enzyme activity, bilirubin levels overlapping with severe GS, and responsiveness to enzyme-inducing agents such as phenobarbital. The global disease burden of CNS remains significant, with considerable variation in clinical outcomes across populations [21]. Distinguishing GS from intermediate UGT1A1 deficiency phenotypes requires careful integration of genotypic, biochemical, and clinical data, reinforcing the diagnostic value of UGT1A1 genotyping in resource-limited settings such as Nepal.

From a clinical standpoint, genetic confirmation of GS facilitates timely diagnostic certainty, obviates unnecessary investigations, enables appropriate patient counseling regarding the benign prognosis, and informs family screening. Although Gilbert Syndrome is a benign genetic variation that does not require special treatment, many patients frequently visit different health facilities for the treatment of jaundice and unnecessarily avoid many foods due to prevailing food taboos, causing mental stress, financial loss, and malnutrition. From a public health perspective, the high prevalence of this pharmacogenetically relevant variant highlights the potential need for development of population-specific clinical practice guidelines for dose modification of UGT1A1-metabolized drugs, implementation of preemptive pharmacogenetic testing protocols, and healthcare provider education regarding the clinical implications of UGT1A1 polymorphisms beyond their association with GS.

## Limitations of the study

The sample size (N = 75) and referral-based recruitment from tertiary centers may introduce selection bias, potentially overrepresenting clinically apparent GS while underrepresenting milder phenotypes. The exclusive focus on c.-3279T > G without concurrent screening for UGT1A1*28, coding region variants (e.g., p.G71R), or other promoter polymorphisms limits comprehensive characterization of the UGT1A1 mutational spectrum. Furthermore, due to financial and technical constraints, Haptoglobin levels, Hemoglobin High-Performance Liquid Chromatography (Hb HPLC), enzymatic analysis of Red Blood Cells (RBCs), membranopathy analysis of the RBC membrane, and evaluation for unstable hemoglobin, could not be performed.

The cross-sectional design precludes longitudinal assessment of temporal bilirubin variability, natural history, or long-term complications. Finally, absence of functional enzyme activity assays prevents direct correlation of genotype with UGT1A1 catalytic efficiency.

Our findings establish a foundation for multicenter, population-based studies with random sampling to validate allele frequencies across Nepal’s ethnically diverse population. Longitudinal cohort studies should assess genotype-stratified outcomes, quality of life, and gallstone risk [22]. Prospective pharmacogenomic investigations examining UGT1A1 variant-associated drug toxicity would enable evidence-based dosing algorithms. Comprehensive mutational screening including UGT1A1*28, coding variants, and haplotype analysis would fully characterize the genetic basis of GS in Nepal. Cost-effectiveness analyses of routine genotyping strategies are essential for healthcare policy formulation in resource-constrained settings.

## Conclusion

This study establishes that UGT1A1*60 (c.-3279T > G) polymorphism is the predominant genetic determinant of Gilbert Syndrome in Nepal, with a G allele frequency of 81.3% with among the highest globally reported cases. The genotype-phenotype correlation, systematic hemolytic exclusion, and preserved hepatic function concludes GS to be a benign inherited disorder of bilirubin conjugation in this cohort. These findings have critical implications for diagnostic algorithms, necessitating prioritization of c.-3279T > G screening in South Asian populations, and for pharmacogenetic risk stratification, given the high prevalence of this variant affecting metabolism of clinically important medications. Integration of UGT1A1 genotyping into routine clinical practice, coupled with development of population-specific pharmacogenetic guidelines, represents an essential advancement toward precision medicine in Nepal. Further multicenter investigations are imperative to validate these findings across Nepal’s diverse ethnic groups and to establish evidence-based frameworks for genotype-guided therapy.
